# Balloon catheter-assisted rescue for misplacement of lumen-apposing stent across the pylorus in endoscopic ultrasound-guided transduodenal drainage of walled-off necrosis

**DOI:** 10.1055/a-2361-4468

**Published:** 2024-08-07

**Authors:** Min Jae Yang, Woohyun Cho, Jae Chul Hwang, Byung Moo Yoo, Jin Hong Kim, Eun Ji Shin

**Affiliations:** 1 1500 Division of Gastroenterology and Hepatology, Department of Medicine, The Johns Hopkins University School of Medicine, Baltimore, United States; 2 37977 Department of Gastroenterology, Ajou University School of Medicine, Suwon, Korea (the Republic of); 3 37977 Medical Information and Media Center, Ajou University School of Medicine, Suwon, Korea (the Republic of)

A 57-year-old man presented with a walled-off necrosis after endoscopic papillectomy. Endoscopic ultrasound-guided transduodenal drainage was attempted using a lumen-apposing metal stent (SPAXUS; TaeWoong Medical, Gimpo-si, South Korea) 1 . The proximal flange was deployed in the necrotic cavity and an enteral flange was deployed within the working channel of the echoendoscope. The stent was ejected from the working channel by pushing the stent delivery system. However, the enteral flange was positioned in the gastric antrum across the pyloric ring because the endoscope tip in the duodenum moved backward into the gastric antrum due to the reaction force generated by pushing the stent delivery system to eject the intrascope channel stent portion ( Fig. 1 , Fig. 2 **a** ).

**Fig. 1:**
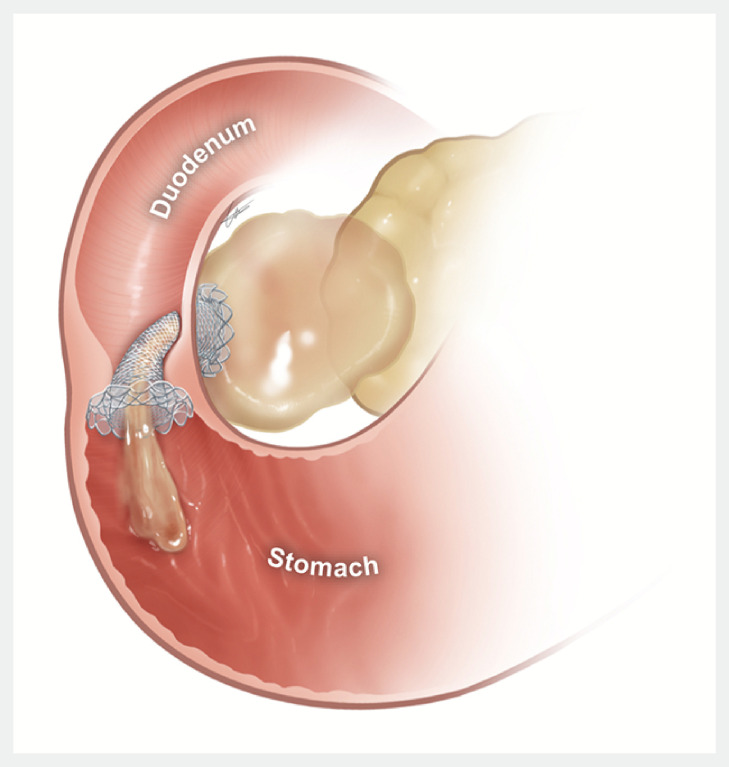
Immediate misplacement of a lumen-apposing metal stent into the gastric antrum across the pyloric ring in endoscopic ultrasound-guided transduodenal drainage of walled-off necrosis. Source: Ajou University School of Medicine.

**Fig. 2:**
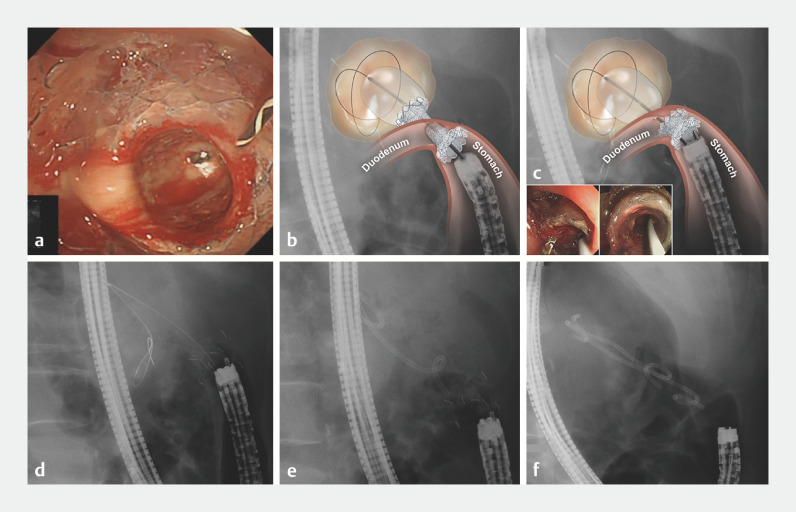
Retrieval balloon catheter-assisted rescue technique for misplacement of a lumen-apposing metal stent during endoscopic ultrasound-guided transduodenal walled-off necrosis drainage. **a** The enteral flange was positioned in the gastric antrum across the pyloric ring. **b** A double-channel gastroscope was introduced, and a balloon catheter inflated after insertion into the necrotic cavity through one channel. **c** Forceps introduced through the second channel of the gastroscope grasped the stent and pulled it out over the balloon catheter until its proximal flange was positioned in the antrum. The inflated balloon was kept in the cavity to secure the fistula tract. **d** The balloon catheter was retrieved with the guidewire remaining in the cavity. **e** A rescue double plastic stent was advanced over the guidewire and placed. **f** An additional plastic stent was inserted alongside the first. Source for graphical illustrations: Source: Ajou University School of Medicine.

A double-channel gastroscope (GIF-2T240; Olympus, Tokyo, Japan) was introduced, and a balloon catheter (Quattro; Cook Medical, Bloomington, Indiana, USA) was inflated after insertion into the necrotic cavity through one channel ( Fig. 2 **b** ). Forceps introduced through the second channel of the gastroscope were used to grasp the stent and pull it out over the balloon catheter until its proximal flange was positioned in the antrum, while the inflated balloon was kept in the cavity to secure the fistula tract ( Fig. 2 **c** ). The balloon catheter was then retrieved with the guidewire remaining in the cavity ( Fig. 2 **d** ). A rescue double plastic stent was advanced over the guidewire and placed ( Fig. 2 **e** ). The lumen-apposing metal stent in the antrum was retrieved from the patient’s mouth. Finally, an additional plastic stent was inserted alongside the first ( Fig. 2 **f** , Video 1 ).

Lumen-apposing metal stent misplacement treated with the retrieval balloon catheter-assisted rescue technique. Source for graphical illustrations: Source: Ajou University School of Medicine.Video 1

When the duodenal puncture site is close to the pyloric ring during endoscopic ultrasound-guided transduodenal intervention, the echoendoscope tip should be adequately supported to ensure that it does not retract into the gastric antrum while ejecting the intrascope channel stent portion. Additionally, the echoendoscope should be twisted in the duodenal bulb to secure adequate space for stent positioning.

Endoscopy_UCTN_Code_CPL_1AL_2AD

## Notes

Endoscopy E-Videos https://eref.thieme.de/e-videos
*E-Videos* is an open access online section of the journal *Endoscopy* , reporting on interesting cases and new techniques in gastroenterological endoscopy. All papers include a high-quality video and are published with a Creative Commons CC-BY license. Endoscopy E-Videos qualify for HINARI discounts and waivers and eligibility is automatically checked during the submission process. We grant 100% waivers to articles whose corresponding authors are based in Group A countries and 50% waivers to those who are based in Group B countries as classified by Research4Life (see: https://www.research4life.org/access/eligibility/ ). This section has its own submission website at https://mc.manuscriptcentral.com/e-videos .
